# The Antiobesity Effects of Buginawa in 3T3-L1 Preadipocytes and in a Mouse Model of High-Fat Diet-Induced Obesity

**DOI:** 10.1155/2019/3101987

**Published:** 2019-07-30

**Authors:** Yea-Jin Park, Dong-Wook Seo, Jae-Yun Ju, Yun-Yeop Cha, Hyo-Jin An

**Affiliations:** ^1^Department of Pharmacology, College of Korean Medicine, Sangji University, Wonju, Gangwon-do 26339, Republic of Korea; ^2^Department of Rehabilitative Medicine of Korean Medicine and Neuropsychiatry, College of Korean Medicine, Sangji University, Wonju, Gangwon-do 26339, Republic of Korea

## Abstract

There has been a remarkable interest in finding lipid inhibitors from natural products to replace synthetic compounds, and a variety of oriental medicinal herbs are reported to have biological activity with regard to lipid inhibition. Buginawa (Bugi) is a novel combined formula that contains twelve medicinal herbs with potential for weight loss induction. We hypothesized that Bugi may have antiobesity effects in 3T3-L1 preadipocytes and in a high-fat diet- (HFD-) induced mouse model. In this study, 3T3-L1 cells were treated with varied concentrations of Bugi (62.5, 125, or 250 *μ*g/mL). Bugi treatment inhibited adipocyte differentiation by suppressing adipogenic transcription genes, including peroxisome proliferator-activated receptor *γ* protein (PPAR*γ*), CCAAT/enhancer-binding protein *α* (C/EBP*α*), sterol regulatory element-binding protein 1 (SREBP1), and CCAAT/enhancer-binding protein *β* (C/EBP*β*). Mice were fed a normal diet or an HFD for 11 weeks, and Bugi was simultaneously administered at 50 or 100 mg/kg. Bugi administration significantly reduced body weight gain and white adipose tissue (WAT) weight and effectively inhibited lipid droplet accumulation in epididymal white adipose tissue (eWAT) and liver tissue. Further, Bugi treatment suppressed mRNA levels of* PPARγ*,* C/EBPα*, and* SREBP1* in eWAT and liver tissue. Our findings demonstrate that Bugi could be an effective candidate for preventing obesity and related metabolic disorders.

## 1. Introduction

Obesity is known to be a major risk factor for a wide range of noncommunicable diseases including chronic obstructive sleep apnea, type 2 diabetes mellitus (T2DM), and various types of cancers [1]. The incidence of obesity is 10.7% in China, 12.8% in the European Union, and 30.4% in the United States [2]. Approximately 70% of American adults are overweight or obese with a BMI ≥ 25 [3]. Obesity is an immoderate accumulation of adipose tissue, an organ largely composed of fat cells. Expansion of adipose tissue is associated with hyperplasia, an increase in cell number, or hypertrophy, an increase in cell size [4]. 3T3-L1 cells are used extensively as a cell culture model to study the molecular control of adipogenesis [5]. Adipocyte differentiation, also known as adipogenesis, is a process in which fibroblast-like preadipocytes develop into mature adipocytes [6]. The cells display an exponential growth phase until confluence is reached, undergo “mitotic clonal expansion (MCE)” that enables an increase in the final proportion of differentiated fat cells, and enter the terminal maturation process of acquiring all the specialized equipment of adipocytes [7]. At the early stage of adipogenesis, transcription factors, including CCAAT/enhancer-binding protein *β* (C/EBP*β*), are induced, thereby stimulating the expression of peroxisome proliferator-activated receptor *γ* protein (PPAR*γ*) and CCAAT/enhancer-binding protein *α* (C/EBP*α*) [8]. PPAR*γ* and C/EBP*α* are regarded as the two main adipogenic transcription factors in this network, positively regulating each other's expression and acting together to control adipogenesis [9]. Additionally, differentiation is enhanced by the transcription factor sterol regulatory binding protein 1 (SREBP1), which is promoted by PPAR*γ* and controls lipogenic factors involved in fatty acid synthesis [10]. Hence, it is very important to modulate the gene expression of PPAR*γ*, C/EBP*α*, and SREBP1 in the study of obesity, since the differentiation process is controlled by these three key adipogenic factors.

Buginawa (Bugi) is a novel combined water extract formula for treating obesity, composed of twelve medicinal herbs (*Cucurbita moschata* Duchesne,* Angelica gigas* Nakai,* Cnidium officinale *Makino,* Poria cocos* Wolf,* Glycyrrhiza uralensis* Fisch.,* Zingiber officinale* Rosc.,* Lycium chinense* Mill.,* Chaenomeles sinensis*,* Acanthopanax sessiliflorus*,* Atractylodes chinensis* Koidz.,* Citrus unshiu* Markovich, and* Zizyphus jujuba*). The major component of Bugi,* C. moschata* Duchesne, also known as pumpkin, helps improve lipid profile, insulin resistance, and other related problems in obese rats [11]. Ui-Jin et al. reported that* A. gigas* Nakai alleviates hyperglycemia and hepatic steatosis in C57BL/KsJ-*db/db* mice [12]. The herbal extract powder containing* C. unshiu* Markovich, YY-312, decreases body fat in overweight adults [13] and saponin-rich fraction of* A. sessiliflorus* inhibits body weight gain in high-fat diet- (HFD-) induced mice without symptoms of diarrhea [14]. Dehydrotramete-nolic acid, isolated from the dried sclerotia of* P. cocos* Wolf, has insulin-sensitizing activity in ST13 preadipocytes [15] and the chloroform fraction of* Z. jujuba* represses adipogenesis in 3T3-L1 preadipocytes [16]. Previous studies have shown that* C. officinale *Makino has anticancerous effects in liver [17], colorectal [18], and oral tissues [19]. The triterpenoid saponins in* G. uralensis* Fisch. and* L. chinense* Mill. leaves have shown antioxidant activity [20, 21], and* Z. officinale* Rosc. (ginger) and* C. sinensis* have anti-inflammatory properties [22, 23]. Here, based on the attributes of each herb, we developed a new formula for treating obesity and examined its effects* in vitro *and* in vivo*.

## 2. Materials and Methods

### 2.1. Chemicals and Reagents

3-Isobutyl-1-methylxanthine (IBMX), dexamethasone (DEX), insulin, and Oil Red O powder were purchased from Sigma-Aldrich Co. LLC (St. Louis, MO, USA). Dulbecco's modified Eagle's medium (DMEM), bovine serum (BS), fetal bovine serum (FBS), and Antibiotic-Antimycotic (ABAM) were purchased from Life Technologies, Inc. (Grand Island, NY, USA). Orlistat was purchased from Tokyo Chemical Inc. (Tokyo, Japan). HFD (30%) was obtained from Research Diets (New Brunswick, NJ, USA).* PPARγ*,* C/EBPα*,* SREBP1*, and glyceraldehyde-3-phosphate dehydrogenase (*GAPDH*) oligonucleotide primers were purchased from Bioneer Corporation (Daejeon, Republic of Korea), and SYBR Premix Ex Taq was purchased from Takara Bio Inc. (Otsu, Japan). Antibodies against PPAR*γ* (cat. No. sc-7273), C/EBP*α* (cat. No. sc-9314), SREBP1 (cat. No. sc-13551), C/EBP*β* (cat. No. sc-150), and *β*-actin (cat. No. sc-81178) were purchased from Santa Cruz Biotechnology, Inc. (Dallas, TX, USA). Horseradish peroxidase conjugated secondary antibodies were purchased from Jackson ImmunoResearch Laboratories, Inc. (West Grove, PA, USA).

### 2.2. Preparation of Bugi

Bugi contained* C. moschata* Duchesne (1 kg),* A. gigas* Nakai (160 g),* C. officinale *Makino (100 g),* P. cocos* Wolf (80 g),* G. uralensis* Fisch. (40 g),* Z. officinale* Rosc. (120 g),* L. chinense* Mill. (60 g),* C. sinensis* (60 g),* A. sessiliflorus* (40 g),* A. chinensis* Koidz. (80 g),* C. unshiu* Markovich (60 g), and* Z. jujuba* (80 g). The twelve herbs were acquired from Nanum Pharmaceutical Company (Seoul, Republic of Korea). Herbs were extracted in water at 99°C for 3 h. The extract was then freeze-dried and the yield was calculated at 33.73% (33.73 g per 100 g of liquid extract). The powder was dissolved in phosphate-buffered saline (PBS, pH = 7.4) and distilled water for* in vitro* and* in vivo *analysis, respectively, and residual powder was stored at −20°C.

### 2.3. Cell Culture and Differentiation of 3T3-L1 Preadipocytes

3T3-L1 preadipocytes were obtained from the Korean Cell Line Bank (Seoul, Republic of Korea). Cells were cultured in DMEM with 10% BS, 1% ABAM, 1 g/L HEPES, and 1.5 g/L sodium bicarbonate and maintained in a humidified atmosphere of 5% CO_2_ at 37°C. For induction of adipocyte differentiation, cells were seeded at a density of 2 × 10^5^ per well into 6-well plates. Two days after cells reached confluence (defined as day 0), cells were differentiated with MDI medium containing 0.5 mM IBMX, 1 *μ*M DEX, and 1 *μ*g/mL insulin. After two days (Day 2), fresh differentiation medium containing insulin was treated for two days (Day 4), and then the medium was changed every two days until days 6-8.

### 2.4. MTT Assay for Cell Viability

3T3-L1 preadipocytes (1 × 10^4^ cells per well) were seeded on 96-well plates overnight and treated with Bugi (7.81 to 500 *μ*g/mL) for 48 h. The 3-(4,5-dimethylthiazol-2-yl)-2,5-diphenyl tetrazolium bromide (MTT) solution (5 mg/mL) was added and cells were incubated at 37°C for 4 h. After removal of the supernatant, 100 *μ*L of DMSO was added to dissolve formazan crystals, and the MTT-formazan product was measured using an Epoch® microvolume spectrophotometer (Bio Tek Instruments Inc., Winooski, VT, USA) at 570 nm.

### 2.5. Oil Red O Staining

Differentiated 3T3-L1 adipocytes (Day 8) were washed three times with PBS and fixed with 10% formaldehyde in PBS at 25°C for 1 h. After fixation, cells were washed three times with distilled water and then stained with Oil Red O working solution (3 mg/mL ORO in 60% isopropanol) at 25°C for 2 h. Cells were rinsed three times with distilled water and photographed with an Olympus SZX10 microscope (Tokyo, Japan). The Oil Red O dye was eluted by isopropanol to determine the intracellular lipid content and was measured with an Epoch® microvolume spectrophotometer at 520 nm.

### 2.6. Western Blot Analysis

Western blot analysis was performed as previously described [24]. In brief, the cells were suspended in PRO-PREP™ protein extraction solution (Intron Biotechnology, Seoul, Republic of Korea). Equal amounts (20 *μ*g) of protein sample were separated on a sodium dodecyl sulfate (SDS) polyacrylamide gel, followed by being transferred onto a polyvinylidene fluoride (PVDF) membrane. Membranes were incubated overnight with primary antibody and then incubated with horseradish peroxidase-conjugated secondary antibody for 2 h. The blots were again washed three times with T/TBS and then visualized by enhanced chemiluminescence (GE Healthcare, Waukesha, WI, USA).

### 2.7. HFD-Induced Obesity Mouse Model

Under approval by the Ethical Committee for Animal Care and the Use of Laboratory Animal of Sangji University (reg. no. 2017-12), eight-week-old male C57BL/6N mice (20 ± 2 g) were used in these animal experiments. Mice were purchased from Daehan Biolink (Daejeon, Republic of Korea) and maintained at 22 ± 2°C and 55 ± 9% humidity, under a 12 h light/dark cycle. Prior to the start of the experiment, mice were acclimated to the modified conditions for 1 week. Mice were weighed and divided into six groups (n = 6 per group) as follows: normal diet group (CON), 30% HFD, HFD + oral administration of orlistat groups (orlistat 10 or 20 mg/kg), and HFD + oral administration of Bugi groups (Bugi 50 or 100 mg/kg). With the exception of the CON group, all other mice were fed an HFD. Bugi- or orlistat-treated groups were administered Bugi or orlistat orally, whereas other groups were treated with physiological saline. Mice were provided water and diet ad libitum for 11 weeks. Body weight and food intake were recorded every week. At the end of the 11-week period, all animals were fasted for 12 h, anaesthetized with Zoletil 50 (20 mg/kg) administered intraperitoneally according to the manufacturer's instruction (Virbac, Carros Cedex, France), and euthanized by cervical dislocation. Liver and adipose tissues were then taken, rinsed, weighed, and directly stored at −80°C until further analysis.

### 2.8. Serum Analysis

At the end of each experiment, blood samples of treated mice were collected and centrifuged at 1000 ×g for 20 min. Serum concentration was used to determine blood urea nitrogen (BUN), aspartate aminotransferase (AST), and alanine aminotransferase (ALT) using enzymatic methods with commercially available kits (BioVision; Milpitas, CA, USA).

### 2.9. Histological Analysis

Liver and epididymal adipose tissues from a representative mouse in each group were fixed in 10% buffered formalin, embedded in paraffin, and cut into 8-*μ*m-thick sections. Some sections were stained with hematoxylin and eosin (H&E) for histological examination of lipid droplets and images were acquired using an Olympus SZX10 microscope.

### 2.10. Quantitative Real-Time Polymerase Chain Reaction (qRT-PCR) Analysis

qRT-PCR analysis was performed as previously described [25]. In brief, the liver and epididymal WAT were homogenized, and total RNA was isolated using the Easy-Blue® reagent. The total RNA was converted to cDNA using a high-capacity cDNA reverse transcription kit (Applied Biosystems; Foster City, CA, USA) and thermocycler (Gene Amp® PCR system 9700; Applied Biosystems). Real-time PCR analysis was conducted using a Step One Plus® Real-time PCR system (Applied Biosystems). Oligonucleotide primers for mouse* PPARγ* were 5′ATCGAGTGCCGAGTCTGTGG3′ (forward) and 5′ GCAAGGCACTTCTGAAACCG3′ (reverse); for mouse* C/EBPα* were 5′ GGAACTTGAAGCACAATCGATC3′ (forward) and 5′ TGGTTTAGCATAGACGTGCACA3′ (reverse); for mouse* SREBP1* were 5′ ATCGCAAACAAGCTGACCTG3′ (forward) and 5′ AGATCCAGGTTTGAGGTGGG3′ (reverse); for mouse* GAPDH* were 5′ GACGGCCGCATCTTCTTGT3′ (forward) and 5′ CACACCGACCTTCACCATTTT3′ (reverse). Gene expression was determined according to the comparative threshold cycle (Ct) method.

### 2.11. Statistical Analysis

Each result is expressed as the mean ± standard deviation (SD) of triplicate experiments. Statistical analysis was performed using SPSS statistical analysis software (version 19.0; International Business Machines, Armonk, NY, USA). Statistically significant differences were determined using analysis of variance and Dunnett's post hoc test, and P-values of less than 0.05 were considered statistically significant.

## 3. Results

### 3.1. Bugi Had Strong Antiadipogenic Effects on 3T3-L1 Preadipocytes

**Figure 1:**
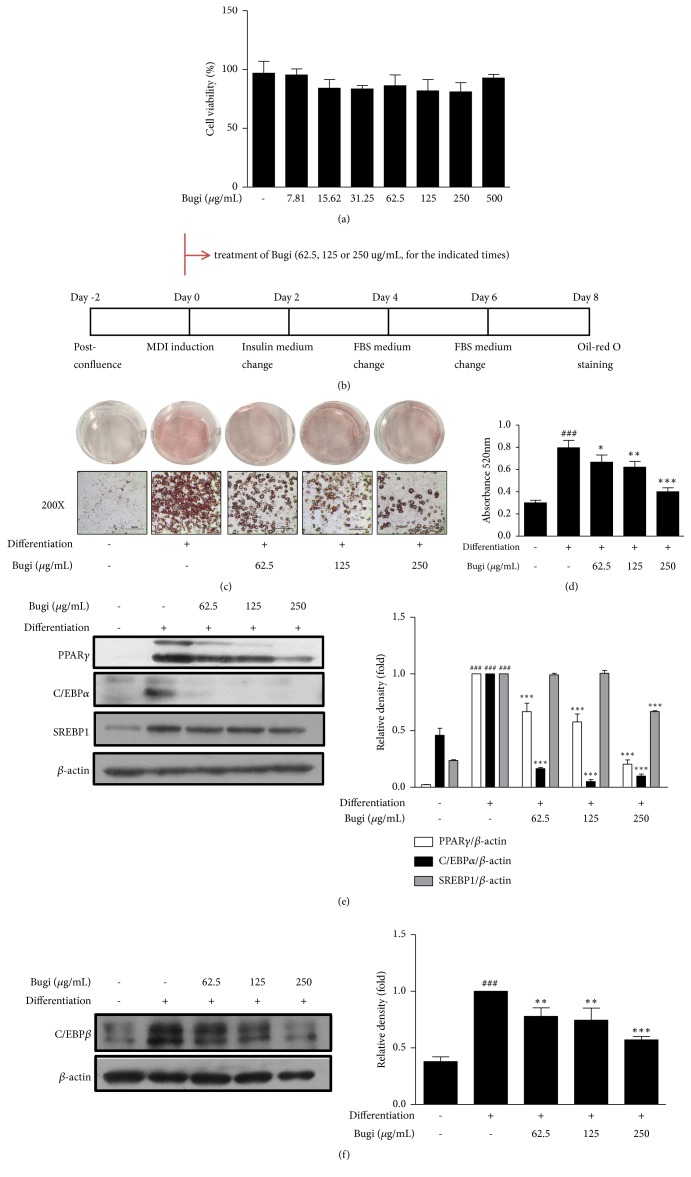
*Bugi inhibits the adipocyte differentiation on 3T3-L1 cells*. (a) The cell viability of Bugi on 3T3-L1 adipocytes. (b) The time scheme of Oil Red O staining. (c) The postconfluent 3T3-L1 cells were differentiated in absence or presence of Bugi (62.5, 125, or 250 *μ*g/mL). The lipid accumulation was measured by Oil Red O staining. (d) The lipid content was quantified by measuring absorbance. (e) The protein expression of PPAR*γ*, C/EBP*α*, SREBP1, and (f) C/EBP*β* was measured on Day 2 or Day 4 after the stimulation of 3T3-L1 differentiation, respectively. Densitometric analysis was performed using ImageJ ver. 1.50i. The values represent the mean ± S.D. ^###^p < 0.001 vs. the control cells;  ^*∗*^p < 0.05,  ^*∗∗*^p < 0.01, and  ^*∗∗∗*^p < 0.001 vs. the differentiated cells.

For MTT assay, cells were treated with various concentrations of Bugi for 48 h. As shown in Figure 1(a), cell viability was unaffected by Bugi at concentrations of up to 500 *μ*g/mL. Timescale of 3T3-L1 cell differentiation is shown in Figure 1(b). Bugi significantly (*p* < 0.05) suppressed lipid accumulation in a dose-dependent manner compared to untreated differentiated cells (Figures 1(c) and 1(d)). Expression of PPAR*γ*, C/EBP*α*, and SREBP1 was measured on Day 4 after the stimulation of 3T3-L1 differentiation. Bugi dramatically (*p* < 0.001) reduced the expression of PPAR*γ* and C/EBP*α*, and a high concentration of Bugi also greatly (*p* < 0.001) reduced SREBP1 expression compared to untreated differentiated cells (Figure 1(e)). As shown in Figure 1(f), Bugi also significantly (*p* < 0.01) decreased C/EBP*β* expression in a dose-dependent manner compared with untreated differentiated cells.

### 3.2. Bugi Reduced Body Weight and Abdominal Fat Content in Mice with HFD-Induced Obesity

**Figure 2:**
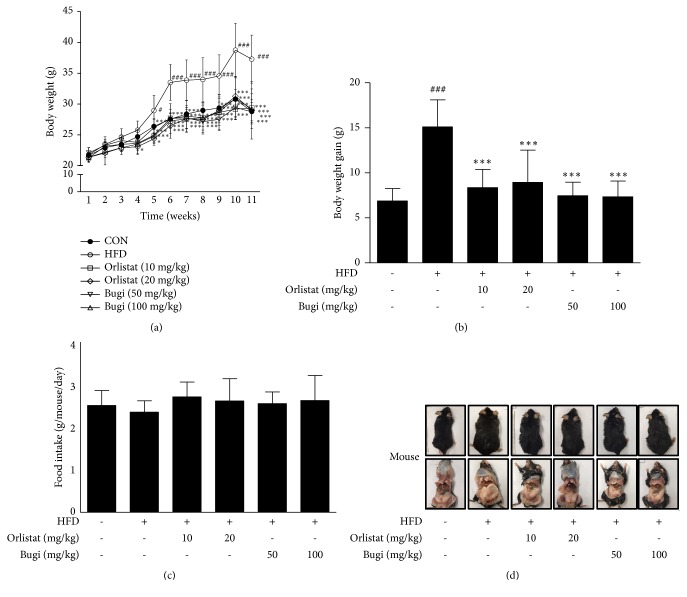
*Bugi inhibits body weight and abdominal fat content in mice with HFD-induced obesity*. (a) Body weight (BW) and (c) food intake were recorded every week. (b) Final body weight gain and (d) abdominal fat content were checked at the end of 11 weeks of experiments. (d) The values represent the mean ± S.D. ^#^p < 0.05 and ^###^p < 0.001 vs. the CON group;  ^*∗*^p < 0.05 and  ^*∗∗∗*^p < 0.001 vs. HFD group.

Body weight was measured weekly, and body weights of the HFD group increased compared to the CON group. However, Bugi significantly inhibited HFD-induced body weight gain. Final body weights of mice fed normal diet, HFD, HFD + orlistat 20 mg/kg, and HFD + Bugi 100 mg/kg were 28.78 ± 1.5 g, 37.30 ± 3.9 g, 28.99 ± 4.6 g, and 29.21 ± 3.2 g, respectively (Figure 2(a)). Final weight gains of mice fed normal diet, HFD, HFD + orlistat 20 mg/kg, and HFD + Bugi 100 mg/kg were 6.87 ± 1.38 g, 15.10 ± 3.00 g, 8.94 ± 3.59 g, and 7.35 ± 1.74 g, respectively (Figure 2(b)). Furthermore, there was no significant (*p* > 0.05) difference in food intake among the experimental groups (Figure 2(c)). At the end of 11-week period, an anatomical examination showed that Bugi treatment effectively repressed body fat mass compared to the HFD group (Figure 2(d)). In addition, long-term treatment with Bugi did not seem to induce liver and kidney toxicity (Table 1).

### 3.3. Bugi Reduced Total White Adipose Tissue Mass in Mice with HFD-Induced Obesity

**Figure 3:**
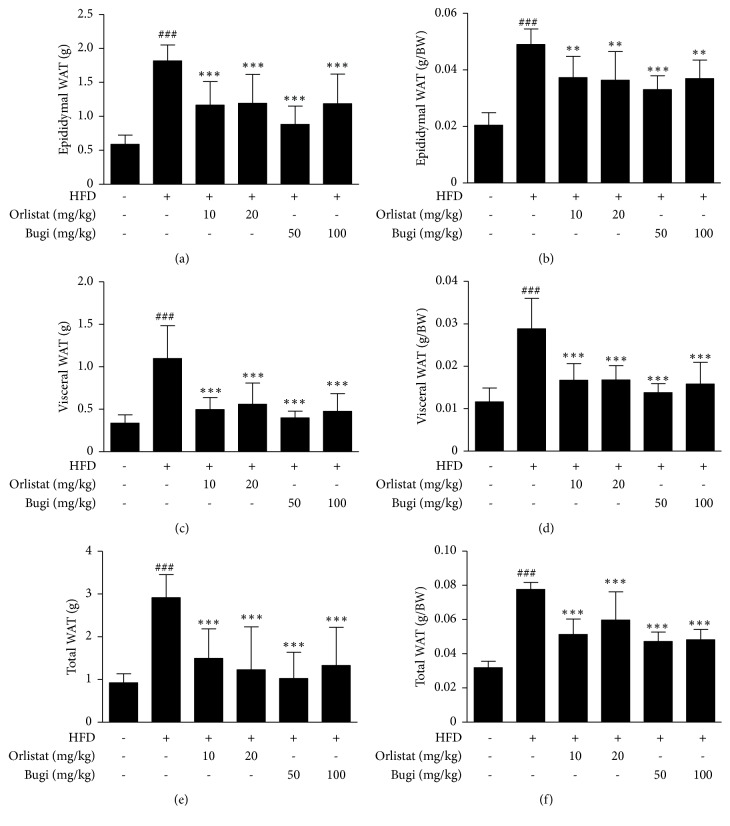
*Bugi inhibits the white adipose tissue (WAT) in mice with HFD-induced obesity*. (a) Epididymal white adipose tissue (eWAT) weight, (b) eWAT with respect to the final BW, (c) visceral adipose tissue weight, (d) visceral adipose tissue weight with respect to the final BW, (e) total adipose tissue weight, and (f) total adipose tissue weight with respect to the final BW were measured at the end of 11 weeks of experiments. The values represent the mean ± S.D. ^###^p < 0.001 vs. the CON group;  ^*∗∗*^p < 0.01 and  ^*∗∗∗*^p < 0.001 vs. HFD group.

As shown in Figure 3, the HFD group showed a remarkable increase in weights of epididymal, visceral, and total WAT compared to the CON group. Bugi- and orlistat-treated groups showed significant (*p* < 0.01) amelioration of the increase in WAT weights compared with the HFD group. The epididymal WAT index (g/BW) of mice fed normal diet, HFD, HFD + orlistat 20 mg/kg, and HFD + Bugi 100 mg/kg was 0.0204 ± 0.0044 g, 0.0489 ± 0.0055 g, 0.0363 ± 0.0101 g, and 0.0368 ± 0.0065 g, respectively (Figure 3(b)). The visceral WAT index of mice fed normal diet, HFD, HFD + orlistat 20 mg/kg, and HFD + Bugi 100 mg/kg was 0.0116 ± 0.0032 g, 0.0288 ± 0.0071 g, 0.0167 ± 0.0033 g, and 0.0158 ± 0.0051 g, respectively (Figure 3(d)). The total WAT index of mice fed normal diet, HFD, HFD + orlistat 20 mg/kg, and HFD + Bugi 100 mg/kg was 0.0318 ± 0.0037 g, 0.0776 ± 0.0041 g, 0.0059 ± 0.0016 g, and 0.0482 ± 0.0059 g, respectively (Figure 3(f)).

### 3.4. Bugi Reduced Adipocyte Size and Expression of Adipogenic-Transcription Genes in Epididymal Adipose Tissue

**Figure 4:**
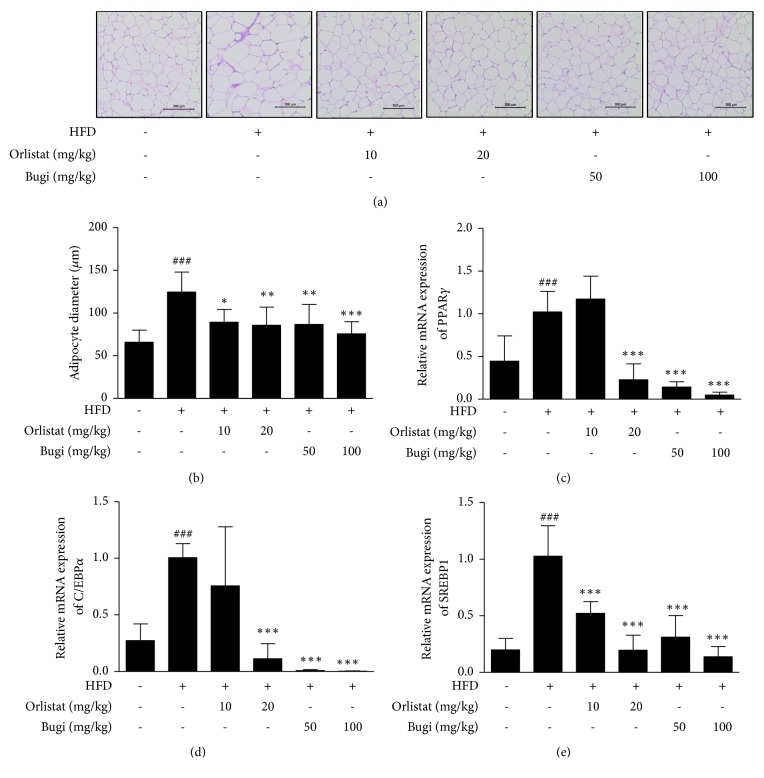
*Bugi inhibits the lipid accumulation and adipogenic transcription factors in eWAT*. (a) The eWAT from representative mice in each group was fixed, embedded in paraffin, and stained with H&E. Images are shown at the original magnification of 100x. (b) The average diameter of adipocytes in eWAT of each group. Total RNA was also prepared from eWAT, and mRNA levels of (c)* PPARγ*, (d)* C/EBPα*, and (e)* SREBP1* were assessed by quantitative RT-PCR (qRT-PCR). The values represent the mean ± S.D. of three independent experiments. ^###^p < 0.001 vs. the CON group;  ^*∗*^p < 0.05,  ^*∗∗*^p < 0.01, and  ^*∗∗∗*^p < 0.001 vs. HFD group. Scale bar is 200 *μ*m.

The HFD group showed larger adipocytes than the CON group. Administration of Bugi significantly reduced abnormal lipid accumulation in epididymal adipose tissue (Figure 4(a)). H&E staining also showed that HFD was associated with an increased average adipocyte diameter in epididymal adipose tissue, compared to an average adipocyte diameter in CON group, whereas the Bugi-treated groups effectively (*p* < 0.01) reduced adipocyte enlargement (Figure 4(b)). Results of qRT-PCR showed significantly enhanced mRNA levels of* PPARγ*,* C/EBPα*, and* SREBP1* in HFD-fed mice. However, Bugi-treated mice had notably (*p* < 0.001) reduced* PPARγ*,* C/EBPα*, and* SREBP1* mRNA levels in epididymal adipose tissue (Figures 4(c), 4(d), and 4(e)).

### 3.5. Bugi Reduced HFD-Induced Morphology Change and Adipogenic-Transcription Gene Expression in Liver Tissue

**Figure 5:**
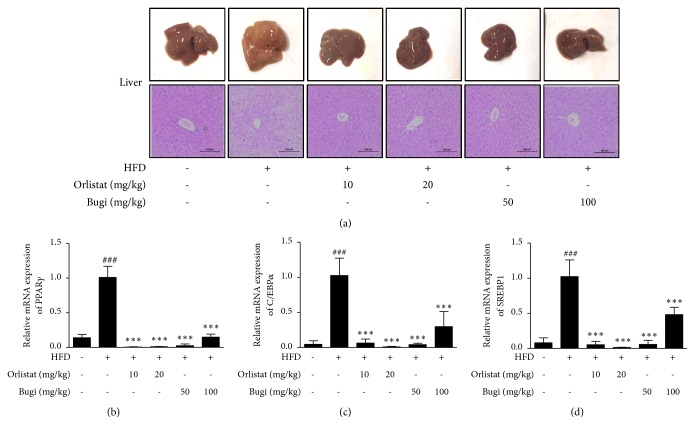
*Bugi inhibits the lipid accumulation and adipogenic transcription factors in liver tissue*. (a) Macroscopic analysis of mouse liver tissue, and the liver tissue from representative mice in each group was fixed, embedded in paraffin, and stained with H&E. Images are shown at the original magnification of 200x. Total RNA was also prepared from liver, and mRNA levels of (b)* PPARγ*, (c)* C/EBPα*, and (d)* SREBP1* were assessed by quantitative RT-PCR (qRT-PCR). The values represent the mean ± S.D. of three independent experiments. ^###^p < 0.001 vs. the CON group;  ^*∗∗∗*^p < 0.001 vs. HFD group. Scale bar is 100 *μ*m.

Gross examination of the 11-week-treated HFD group showed livers with markedly lightened coloring and increased size compared to the CON group. Oral administration of Bugi (50 or 100 mg/kg) significantly recovered the HFD-induced white-colored fatty liver. In addition, a histological examination of liver sections stained with H&E showed that Bugi significantly reduced the HFD-induced enlargement of lipid droplets (Figure 5(a)). As shown in Figure 5(b), there were significant differences in* PPARγ*,* C/EBPα*, and* SREBP1* hepatic mRNA levels between the CON and HFD groups. Levels of* PPARγ*,* C/EBPα*, and* SREBP1* mRNA were significantly (*p* < 0.001) downregulated in the liver of Bugi-treated groups compared with those in the HFD group.

## 4. Discussion

Obesity is becoming one of the most prevalent health problems across all age groups worldwide, resulting in a notable increase in mortality and morbidity related to T2DM, coronary heart diseases, metabolic syndrome, stroke, and cancers [26]. Since weight loss is universally highly difficult to achieve, and particularly challenging to maintain, there have been numerous efforts to develop adjunctive pharmacological approaches over many decades [27]. Orlistat and sibutramine, representative antiobesity drugs, are currently available, but they are expensive and have potentially dangerous side effects [28]. Therefore, there has been a keen interest in finding lipid inhibitors from natural products to replace synthetic compounds, and natural substances are presumed to be safe. Specifically, a variety of oriental medicinal herbs are reported to have biological activity to reduce weight gain [29].

Bugi contains* C. moschata* Duchesne,* A. gigas* Nakai,* C. officinale* Makino,* P. cocos* Wolf,* G. uralensis* Fisch.,* Z. officinale* Rosc.,* L. chinense* Mill.,* C. sinensis*,* A. sessiliflorus*,* A. chinensis* Koidz.,* C. unshiu* Markovich, and* Z. jujuba*. Previous studies indicate that* C. moschata* Duchesne,* A. gigas* Nakai,* A. sessiliflorus*,* P. cocos* Wolf, and* Z. jujube* might be effective in treating obesity [11–16].* G. uralensis* Fisch.,* L. chinense* Mill.,* Z. officinale* Rosc., and* C. sinensis* have antioxidant or anti-inflammatory effects [20–23]. Another study suggested that antioxidant natural products can be regarded as an effective medication for weight loss and obesity treatment [28]. Therefore, we developed a new prescription named Buginawa for inducing weight loss. The aim of this study was to determine the antiobesity effects of Bugi* in vitro* and* in vivo*.

Adipocytes are derived from mesenchymal stromal cells (MSCs), which are precursor cells that can differentiate to several lineages (e.g., osteocytes, chondrocytes, and adipocytes) [30]. 3T3-L1 fibroblasts are precursor cells used as a model to assess adipogenesis. Therefore, we investigated whether Bugi regulates adipocyte differentiation by inhibiting the transcription genes using 3T3-L1 cells. We first examined effect of Bugi on cell viability in 3T3-L1 preadipocytes. Previous studies using herbal formula have set highest concentrations of those at 500 or 1000 ug/mL to determine cytotoxicity in 3T3-L1 cells [31–34]. As shown in Figure 1(a), a slight reduction in cell viability was noticeable as the concentration increases, but it was clearly shown that the cell viability was over 80%. Therefore, concentrations of 62.5, 125, and 250 *μ*g/mL of Bugi, which are noncytotoxic dose, were used for* in vitro *experiments. Then, we examined the effect of Bugi on intracellular lipid accumulation in mature 3T3-L1 adipocytes using Oil Red O staining. Oil Red O staining showed that Bugi decreased lipid droplets in a dose-dependent manner (Figures 1(c) and 1(d)). To understand the molecular mechanisms underlying Bugi's antiadipogenic effect, the expression of key transcription factors related to adipocyte differentiation was evaluated. During this process, the expression of adipogenic transcription factors, including PPAR*γ*, C/EBP*α*, and SREBP1 is stimulated [35]. C/EBP*β* is expressed earlier during adipogenesis because it is required for the induction of MCE caused by MDI medium [36] and then triggers the expression of PPAR*γ* and C/EBP*α* in 3T3-L1 cells [37]. The data showed that Bugi markedly suppressed the PPAR*γ*, C/EBP*α*, and SREBP1 expression on Day 4 but also C/EBP*β* expression on Day 2 in differentiated 3T3-L1 cells (Figures 1(e) and 1(f)). Surprisingly, among the transcription factors involved in adipogenesis (PPAR*γ*, C/EBP*α*, SREBP1), Bugi greatly suppressed the C/EBP*α* expression. C/EBP*α* induces many adipocyte genes directly, and* in vivo* studies indicated an important role for this factor in the development of adipose tissue [38], so Bugi could affect the adipocyte gene directly. These data finally suggest that Bugi exhibits antiadipogenesis effect by inhibiting the transcription genes involved in adipocyte differentiation.

C57BL/6 mice have been widely used as a model of human obesity and T2DM since the disease results from an interaction between environmental and genetic factors [39]. The major cause of obesity is energy imbalance owing to excessive caloric consumption that occurs with the intake of an HFD [40]. Orlistat was used as a positive control because it accomplishes weight loss by inducing fat malabsorption and it has a well-established long-term safety profile [27]. However, its efficacy is limited, and several side effects (e.g., fecal incontinence, flatus with discharge, and steatorrhoea) have been reported [41–43]. According to study of dose calculation, therapeutic and 2 therapeutic doses of orlistat are 10.7 and 20 mg/kg in mice, respectively [44]. In addition, several studies on obesity also have used orlistat at 10 to 20 mg/kg [45–51]. Therefore, in our study, orlistat-treated groups were administered orlistat at 10 and 20 mg/kg. On the other hand, previous studies using herbal formula have set doses of those at over 200 mg/kg in C57B/6 mice [32, 52–54]. Considering that Bugi is composed of 12 herbs, the doses have been set at 50 and 100 mg/kg. With the above points in mind, in current study, we examined the antiobesity effects of Bugi in an HFD-induced C57BL/6N mouse model by treating them with doses of 50 and 100 mg/kg/day for 11 weeks. As shown in Figure 2(a), the HFD group had a significantly higher BW, whereas Bugi administration was associated with a significantly lower BW. Final BW gain also decreased by 51.32%, in the Bugi-treated (100 mg/kg) group compared to the HFD group (Figure 2(b)), although total food intake did not differ among groups (Figure 2(c)). In previous study about antiobesity effect of a water-soluble extract of C.* moschata*, body weight of mice administered with extract of C.* moschata* was lower by 18% as compared with that of the HFD group [55]. In this study, the body weight of mice administrated with Bugi was lower by 33% as compared with that of the HFD group. So, we suppose that the effect of Bugi is better than only C.* moschata. *Furthermore, Bugi is made by following the rules for making prescription to maximize the effect in Korean traditional medicine. However, we are going to further study about the comparative effect of C.* moschata *and Bugi. In addition, administration of Bugi to mice had no significant toxic effects on ALT, AST, and BUN; the plasma activities of AST and ALT, and BUN are well-known enzyme parameters of liver and kidney injury, respectively (Table 1).

There is an increasing appreciation for the metabolic activity and endocrine functions of WAT and its important role in the development of various metabolic diseases [56]. In mice, eWAT expansion occurs under an HFD condition, which contributes to the development of metabolic syndromes such as obesity, insulin resistance, T2DM, and cardiovascular disease [57]. Therefore, we examined the mass, histology, and mRNA expression involved in adipogenesis in the eWAT of mice. As shown in Figure 3, Bugi administration dramatically decreased HFD-induced increases in fat mass index (g per BW) in all adipose tissues examined, including epididymal, visceral, and total WAT. The mass of epididymal, visceral, and total WAT declined by 24.74%, 45.14%, and 37.89%, respectively, in the Bugi (100 mg/kg) treated group compared with those of the HFD group. The adipocyte size was also noticeably smaller in Bugi-treated mice than in HFD-fed mice (Figures 4(a) and 4(b)). Some studies showed that PPAR*γ* is important for fat cell development and mice with adipose tissue-specific depletion of PPAR*γ* had decreased fat pad size in adipose tissue and liver [58]. C/EBP*α* is commonly expressed in adipose tissue and liver, and it was reported that absence of C/EBP*α* repress formation of WAT* in vivo *[59]. SREBP1 preferentially activates genes related to lipogenesis and E. Stelmanska et al. reported that SREBP1 is expressed much higher in abdominal WAT (epididymal and perirenal) than subcutaneous adipose tissue [60]. In liver, SREBP1 plays also a major role in the regulation of* de novo* lipogenesis [61]. Bugi treatment resulted in decreased* PPARγ*,* C/EBPα*, and* SREBP1* mRNA levels in eWAT compared to those in the HFD group (Figures 4(c), 4(d), and 4(e)). These results showed that Bugi may ameliorate the accumulation of eWAT by controlling these transcription factors in HFD-induced obese mice.

Defects in fat metabolism may lead to an imbalance in energy consumption and fat combustion, which lead to pathogenesis of hepatic steatosis followed by lipid storage [62]. Our results also showed that the livers of the HFD group were lighter in color and larger in size, which is indicative of liver steatosis. Notably, Bugi administration recovered these changes as evidenced in the livers of the Bugi group, which were dark red, small, and similar to those in the CON group. Hepatocytes of the HFD group had severe macrovascular accumulation of lipid droplets, whereas the hepatocytes of Bugi groups had mild accumulation of lipid droplets (Figure 5(a)). Moreover, Bugi administration dramatically lowered mRNA levels of* PPARγ*,* C/EBPα*, and* SREBP1*, suggesting that Bugi also exhibits a hepatoprotective effect in HFD-induced obese mice.

## 5. Conclusions

**Figure 6:**
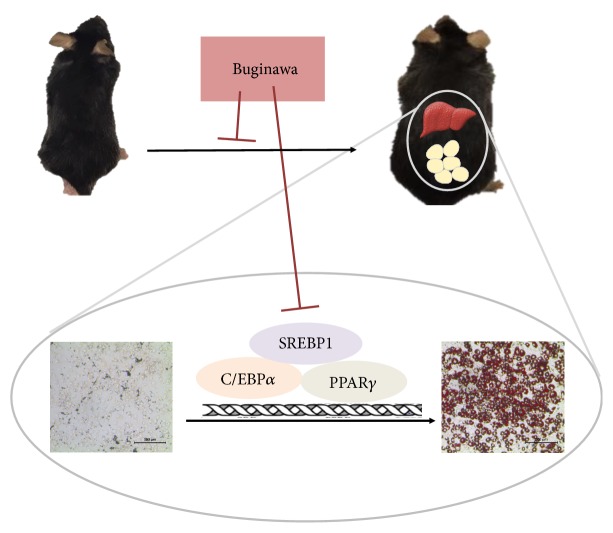
*The mechanism of Bugi*. Bugi treatment effectively reduced lipid accumulation by modulating the levels of PPAR*γ*, C/EBP*α*, and SREBP1 in the eWAT and liver tissue of HFD-induced mice, as well as 3T3-L1 cells.

Here, we demonstrated that Bugi treatment effectively repressed adipogenesis in 3T3-L1 cells, reduced BW and WAT weight in HFD-induced mice, and reduced levels of* PPARγ*,* C/EBPα*, and* SREBP1* in eWAT and liver tissue (Figure 6). Thus, Bugi may be promising natural products for management of weight gain and obesity-associated metabolic disorders.

## Figures and Tables

**Table 1:** Effect of Bugi administration on blood biochemistry in HFD-induced mice.

	Parameters		
Groups	BUN (mg/dL)	AST (U/L)	ALT (U/L)
CON	4.15 ± 0.47	36.86 ± 5.79	7.76 ± 1.83
HFD	3.84 ± 0.26	34.11 ± 9.34	7.24 ± 1.63
Orlistat (10 mg/kg)	3.69 ± 0.23	30.00 ± 1.32	5.05 ± 0.41
Orlistat (20 mg/kg)	3.73 ± 0.24	38.19 ± 5.37	5.26 ± 0.41
Bugi (50 mg/kg)	3.99 ± 0.44	39.50 ± 7.55	5.75 ± 1.16
Bugi (100 mg/kg)	3.98 ± 0.42	36.75 ± 2.22	5.67 ± 1.21

The values are represented as mean ± S.D (n=6). There were no statistically significant differences in the parameters from all groups. Abbreviations: BUN, blood urea nitrogen; AST, aspartate aminotransferase; ALT, alanine aminotransferase.

## Data Availability

The datasets used and/or analyzed in this study are available from the corresponding authors on reasonable request.

## Abbreviations

Bugi: Buginawa
HFD: High-fat diet
WAT: White adipose tissue
eWAT: Epididymal white adipose tissue
PPAR*γ*: Peroxisome proliferator-activated receptor *γ*
C/EBP*α*: CCAT/enhancer-binding protein *α*
SREBP1: Sterol regulatory element-binding protein 1
C/EBP*β*: CCAT/enhancer-binding protein *β*.
